# Disruption of a licorice cellulose synthase-derived glycosyltransferase gene demonstrates its *in planta* role in soyasaponin biosynthesis

**DOI:** 10.1007/s00299-023-03095-6

**Published:** 2023-12-23

**Authors:** Manami Sakanishi, Soo Yeon Chung, Kentaro Fujiwara, Mareshige Kojoma, Toshiya Muranaka, Hikaru Seki

**Affiliations:** 1 Department of Biotechnology, Graduate School of Engineering, Osaka University, 2-1 Yamadaoka, Suita, Osaka 565-0871 Japan; 2 Faculty of Pharmaceutical Sciences, Health Sciences University of Hokkaido, 1757, Kanazawa, Tobetsu, Hokkaido 061-0293 Japan; 3 Industrial Biotechnology Initiative Division, Institute for Open and Transdisciplinary Research Initiatives, Osaka University, 2-1 Yamadaoka, Suita, Osaka 565-0871 Japan

**Keywords:** Biosynthesis, Cellulose synthase-like, Genome editing, Glycosyltransferase, Licorice, Soyasaponin

## Abstract

**Key message:**

**CRISPR–Cas9-mediated disruption of a licorice cellulose synthase-derived glycosyltransferase gene, GuCSyGT, demonstrated the in planta role of GuCSyGT as the enzyme catalyzing 3-O-glucuronosylation of triterpenoid aglycones in soyasaponin biosynthesis.**

**Abstract:**

Triterpenoid glycosides (saponins) are a large, structurally diverse group of specialized metabolites in plants, including the sweet saponin glycyrrhizin produced by licorice (*Glycyrrhiza uralensis*) and soyasaponins that occur widely in legumes, with various bioactivities. The triterpenoid saponin biosynthetic pathway involves the glycosylation of triterpenoid sapogenins (the non-sugar part of triterpenoid saponins) by glycosyltransferases (GTs), leading to diverse saponin structures. Previously, we identified a cellulose synthase-derived GT (CSyGT), as a newly discovered class of triterpenoid GT from *G*. *uralensis*. GuCSyGT expressed in yeast, which could transfer the sugar glucuronic acid to the C3 position of glycyrrhetinic acid and soyasapogenol B, which are the sapogenins of glycyrrhizin and soyasaponin I, respectively. This suggested that GuCSyGT is involved in the biosynthesis of glycyrrhizin and soyasaponin I. However, the *in planta* role of GuCSyGT in saponin biosynthesis remains unclear. In this study, we generated *GuCSyGT*-disrupted licorice hairy roots using CRISPR–Cas9-mediated genome editing and analyzed the saponin content. This revealed that soyasaponin I was completely absent in *GuCSyGT*-disrupted lines, demonstrating the *in planta* role of GuCSyGT in saponin biosynthesis.

**Supplementary Information:**

The online version contains supplementary material available at 10.1007/s00299-023-03095-6.

## Introduction

Triterpenoid saponins are structurally diverse, specialized metabolites in plants containing one or more sugar moieties attached to hydrophobic triterpenoid aglycones, called sapogenins. Triterpenoid saponins have a wide range of biological activities, like glycyrrhizin in the medicinal legume licorice (*Glycyrrhiza uralensis*) and ginsenosides in ginseng (*Panax ginseng*). Several of these compounds are major components of traditional Japanese herbal medicines. Soyasaponins, which are widely found in legumes, affect the functionality and taste of soy-based foods (Yano et al. 2018 and references therein).

**Fig. 1:**
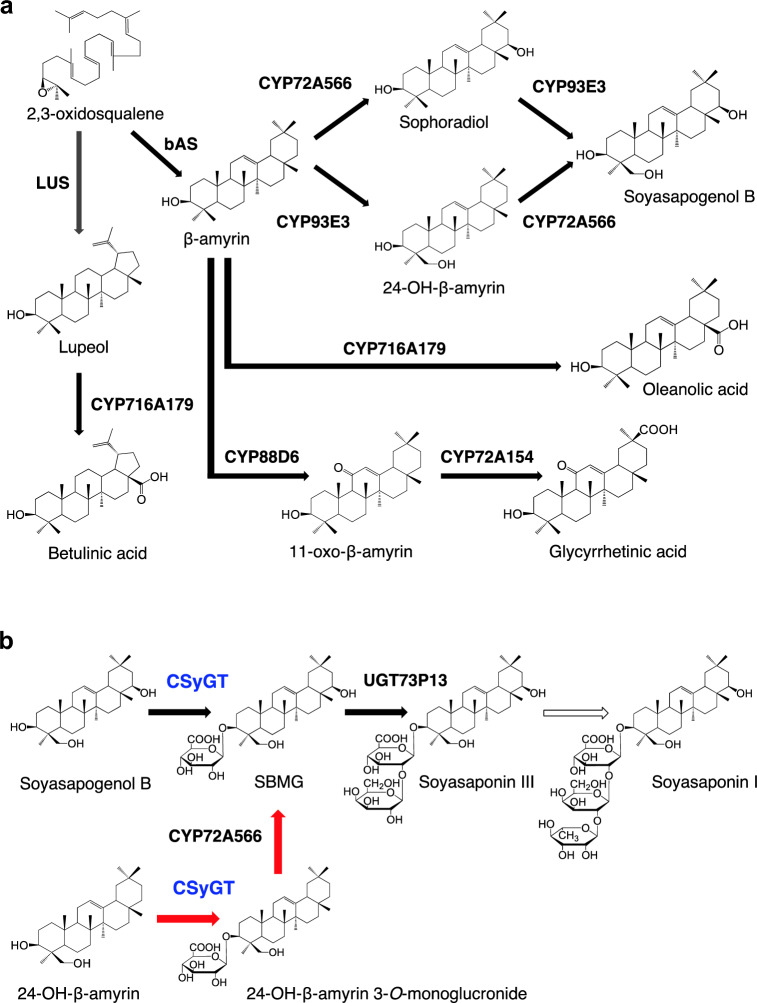
Proposed biosynthetic pathway of triterpenoids in licorice. **a** Pathways for the production of sapogenins. **b** Glycosylation steps in the biosynthesis of soyasaponin I. CSyGT is marked in blue. Black arrows indicate reactions catalyzed by characterized enzymes. Arrow outlines indicate a reaction catalyzed by a yet-uncharacterized enzyme. Red arrows indicate a newly proposed preferred route for soyasaponin biosynthesis *in planta*

The triterpenoid saponin biosynthesis pathway has been studied extensively (reviewed in Thimmappa et al. 2014 and Seki et al. 2015). The first step in the structural diversification of triterpenoid saponins is cyclization of their common precursor 2,3-oxidosqualene, a linear compound of 30 carbon atoms produced via the mevalonate pathway, by a group of enzymes called oxidosqualene cyclases (OSCs). In many cases, the cyclic triterpene scaffold synthesized by OSC undergoes site-specific oxidization by cytochrome P450 monooxygenases (CYPs) to produce sapogenins with various structures. Finally, saponins are synthesized by multiple glycosylation of the sapogenin (Fig. 1). Variation in the number, composition, and position on the triterpene scaffold of sugar chains is thought to affect intra/extracellular transport and storage in plants, as well as biological activity, taste, and bioabsorbability (Bowles et al. 2005). Enzymes called UDP-dependent glycosyltransferases (UGT), characterized by a conserved 44-amino-acid sequence called the plant secondary product glycosyltransferase (PSPG) box, are widely believed to catalyze the glycosylation of various specialized metabolites in plants using a UDP-sugar donor, such as UDP-glucose, UDP-galactose, UDP-arabinose, UDP-rhamnose, UDP-xylose, or UDP-glucuronic acid (Seki et al. 2015).

Licorice, which is one of the most economically important medicinal plants in Japan (Hayashi and Sudo 2009), produces two structurally distinct triterpenoid saponins: glycyrrhizin (its unique active constituent) and soyasaponin I (found widely in legumes). Glycyrrhizin and soyasaponin I share a common biosynthetic intermediate, β-amyrin. Most of the enzymes involved in their biosynthetic pathways have been identified (Hayashi et al. 2001; Seki et al. 2008, 2011; Tamura et al. 2018), including the UGTs UGT73P12, which transfers a glucuronosyl moiety to glycyrrhetinic acid 3-*O*-monoglucuronide, and UGT73P13, which transfers a galactosyl moiety to soyasapogenol B 3-*O*-monoglucuronide (SBMG) as the second sugar moiety at the C3 position in glycyrrhizin and soyasaponin I (Nomura et al. 2019). However, the UGT that catalyzes the transfer of the first conserved glucuronosyl moiety at the C3 position of glycyrrhizin and soyasaponins has long remained unknown. Recently, we identified a cellulose synthase-derived GT (GuCSyGT) as a newly discovered class of triterpenoid GTs from licorice (Chung et al. 2020). The cellulose synthase superfamily consists of the cellulose synthase family (CesA), which catalyzes cellulose biosynthesis, and 10 cellulose synthase-like families (CslA–H, J, and M), which are predicted to be involved in the biosynthesis of hemicellulose. GuCSyGT was classified as a member of cellulose synthase-like subfamily M (CslM), although its biological function is still largely unknown. GuCSyGT, expressed in an engineered yeast (*Saccharomyces cerevisiae*) producing glycyrrhetinic acid or soyasapogenol B endogenously, can transfer glucuronic acid to the C3 position of glycyrrhetinic acid and soyasapogenol B only when co-expressed with UDP-glucose dehydrogenase (UGD) to enable the synthesis of UDP-glucuronic acid from endogenous UDP-glucose in yeast (Chung et al. 2020). These results suggest the involvement of GuCSyGT in the biosynthesis of glycyrrhizin and soyasaponin I. However, the *in planta* function of GuCSyGT in saponin biosynthesis remains unclear.

To confirm the *in planta* contribution of GuCSyGT to saponin biosynthesis, this study generated transgenic hairy roots with a disrupted GuCSyGT gene by CRISPR–Cas9-mediated genome editing. Analysis of the saponin content of these *GuCSyGT*-disrupted hairy roots revealed that soyasaponin I and its biosynthetic intermediates soyasaponin III and SBMG were completely absent, indicating that GuCSyGT plays an essential role in soyasaponin biosynthesis in licorice. Moreover, an analysis of the sapogenin composition revealed that efficient conversion from 24-hydroxy-β-amyrin to soyasapogenol B, catalyzed by CYP72A566 (β-amyrin and 24-hydroxy-β-amyrin C22 hydroxylase) (Tamura et al. 2018), was hampered in *GuCSyGT*-disrupted lines by unknown mechanisms. Possible mechanisms of soyasapogenol B biosynthesis perturbation in *GuCSyGT*-disrupted lines will be discussed herein.

## Materials and methods

### Plant materials

Seeds of *Glycyrrhiza uralensis* Fischer (accession no. GLY-URA-001) were collected in the medicinal plant garden of Health Sciences University of Hokkaido (HSUH), Hokkaido, Japan. A voucher specimen was deposited in the Herbarium of Faculty of Pharmaceutical Sciences, HSUH.

### Cloning of the* GuCSyGT* genomic fragment

Genomic DNA was isolated from a tissue-cultured stolon of *G. uralensis* (Kojoma et al. 2010) using a Nucleon Phytopure Genomic DNA Extraction Kit (GE Healthcare, Chicago, IL, USA) according to the manufacturer’s instructions. PCR was performed using primers 1 and 2 (Supplementary Table S1), which were designed based on the full-length coding sequence of GuCSyGT isolated from *G. uralensis* strain 308–19 (accession no. LC500232). The genomic fragment isolated from strain GLY-URA-001 was cloned into pCR4Blunt-TOPO (Invitrogen, Waltham, MA, USA) and sequenced (accession no. LC778452).

### Chemicals

Authentic standards of β-amyrin, lupeol, oleanolic acid, and glycyrrhetinic acid were purchased from Extrasynthese (Lyon, France). Betulinic acid was purchased from Tokyo Chemical Industry (Tokyo, Japan). Soyasapogenol B was purchased from Tokiwa Phytochemical (Chiba, Japan). Soyasaponins I and III were purchased from ChromaDex (Irvine, CA, USA). Glycyrrhetinic acid was purchased from Nacalai Tesque (Kyoto, Japan). SBMG was isolated from a crude soybean saponin powder (Wako Pure Chemical Industries, Osaka, Japan), described in a previous study (Yano et al. 2018). Sophoradiol and 24-hydroxy-β-amyrin were kindly provided by Dr. Kiyoshi Ohyama.

### Generation of* GuCSyGT*-disrupted hairy root lines

The multiplex guide RNA (gRNA)-expressing CRISPR–Cas9 vector pMgP237-2A-GFP (Hashimoto et al. 2018) was used. The target sequences of the gRNAs (Supplementary Table S2) were selected using the web-based tool CRISPRdirect (https://crispr.dbcls.jp; Naito et al. 2015). Four gRNA target sequences were simultaneously transferred to pMgP237-2A-GFP, generating the *GuCSyGT*-targeting construct, and introduced into *A. rhizogenes* strain ATCC15834. The generation of transgenic hairy roots was performed as described previously (Tamura et al. 2018). Crude genomic DNA was extracted from the induced hairy root lines as described elsewhere (Yasumoto et al. 2020), and mutagenesis was confirmed by PCR using primers 9 and 10 (Supplementary Table S1) and electrophoresis using the MCE-202 MultiNA Microchip Electrophoresis System (Shimadzu, Kyoto, Japan). The amplicons were cloned into pJET1.2/blunt Cloning Vector (Thermo Fisher Science, Waltham, MA, USA), and randomly selected colonies were sequenced. Genome-edited hairy roots were subcultured in liquid half-strength McCown Woody Plant Medium (Duchefa Biochemie, Haarlem, The Netherlands) supplemented with 1% sucrose, 0.01 μM GA_3_, and 125 mg/L of cefotaxime.

### LC–MS analysis

Hairy roots were harvested, lyophilized, and powdered using a multi-beads shocker (Yasui Kikai, Osaka, Japan). Then, 5 mg samples were extracted three times with 1 mL HPLC-grade methanol by 30 s vortex-mixing and 20 min sonication, after which the solution was centrifuged. The resulting supernatant was passed through a GL chromate disk 4A filter (pore size: 0.2 μm; GL Sciences, Tokyo, Japan) to remove debris. The resulting samples were used for LC–MS analysis. Extracted metabolites were detected using the ACQUITY UPLC/MS system (Waters, Milford, MA, USA). To separate compounds, an ACQUITY UPLC BEH C18 column (2.1 × 150 mm; particle size, 1.7 μm; Waters) was used with an ACQUITY TQ detector (Waters) in electrospray ionization negative-ion mode. The mobile phase consisted of 0.05% (v/v) acetic acid in water (solvent 1) and 0.05% (v/v) acetic acid in acetonitrile (solvent 2). The mobile-phase gradient was set according to previous research (Chung et al. 2020), with slight modifications. The sample manager and column were kept at 15 °C and 40 °C, respectively. For MS detection, the capillary voltage was set to 3.0 kV, the source temperature was 120 °C, the desolvation temperature was 350 °C, the cone gas flow was 50 L/h, and the desolvation gas flow was 600 L/h.

### GC–MS analysis

Lyophilized powder (5 mg) was extracted twice with 2 mL methanol/chloroform (1:1, v/v) using a sonication-assisted method. After the solvents were removed, 1 mL methanol and 1 mL 4 M HCl were added to the residue and hydrolyzed at 80 °C for 1 h to remove the sugar moieties of triterpenoid saponins. The hydrolyzed products were extracted twice with 2 mL hexane/EtOAc (1:1, v/v), dried *in vacuo*, and resuspended in 400 μL methanol/chloroform (1:1, v/v). Then, 100 μL solution was dried in a GC–MS vial, resuspended in 50 μL N,N-dimethylformamide, and trimethylsilylated with 50 μL N,O-bis(trimethylsilyl) trifluoroacetamide + trimethylchlorosilane, 99:1 (Sigma-Aldrich, St. Louis, MO, USA), at 80 °C for 30 min before GC–MS analysis.

GC–MS analyses were performed on a 5977A MSD mass spectrometer (Agilent Technologies, Santa Clara, CA, USA) connected to a 7890B gas chromatograph (Agilent Technologies) with an HP-5MS capillary column (30 m × 0.25 mm internal diameter, 0.25 μm film thickness; Agilent Technologies). The injection temperature was set at 250 °C. The column temperature program was as follows: 80 °C for 1 min, followed by an increase to 300 °C at a rate of 20 °C/min, and a hold at 300 °C for 35 min. The carrier gas was helium at a flow rate of 1.0 mL/min. The ion source temperature was 230 °C and the quadrupole temperature was 150 °C. One microliter of the derivatized sample was injected in splitless injection mode. Mass spectra were recorded in the range of 50–750 m*/z*. Peaks were identified by comparing their Rt and mass spectra with those of authentic standards. The concentrations of triterpenoid sapogenins in hairy roots were determined by comparison with authentic standard curves constructed using β-amyrin, 24-hydroxy-β-amyrin, oleanolic acid, betulinic acid, sophoradiol, and soyasapogenol B.

### qPCR

qPCR was performed as described previously (Tamura et al. 2017). The relative transcript level of each target gene was calculated using β-tubulin (Seki et al. 2008) (GenBank accession no. LC318135) as a reference gene. The amplification of each sample was performed three times, with primers 11–18 (Supplementary Table s1). Comparisons between two groups of data were calculated using the Student’s *t* test (*P < 0.05).

## Results

### Genome editing of GuCSyGT in transformed hairy roots

**Fig. 2:**
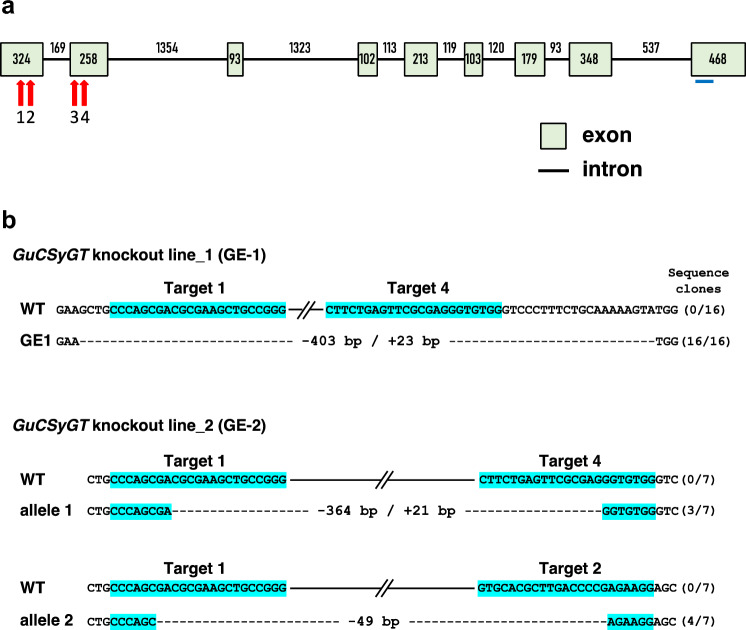
Disruption of the *GuCSyGT* gene in transgenic hairy roots. **a** Genomic structure of *GuCSyGT* showing the length of all exons and introns in base pairs. Red arrows indicate the positions of the target sites (T1–T4). A blue bar indicates the region amplified in the qPCR analysis shown in Fig. 5. **b** Genotyping of the genome-edited lines (GE-1 and GE-2). Dotted lines represent the deleted sequence. Solid lines represent the sequence not fully displayed. The target sequences are highlighted in pale blue

To design the target sequences for CRISPR–Cas9-mediated genome editing, the structure of *GuCSyGT* was determined by genomic PCR amplification and sequencing. The *GuCSyGT* genomic fragment containing the region from the start to the stop codons was PCR-amplified and cloned. Sequencing the 5916 bp fragment obtained from three independent clones and comparison with the corresponding cDNA sequence revealed that the GuCSyGT gene has nine exons (Fig. 2a). Two target sequences in the first exon (T1 and T2), and another two in the second exon (T3 and T4), were selected (Fig. 2a; Supplementary Table S2) using CRISPRdirect software (Naito et al. 2015), and subsequent homology searches against the draft genome assembly of *G*. *uralensis* (Mochida et al. 2016) were done to reduce the risk of off-target effects. These target sequences were simultaneously integrated into the vector pMgP237-2A-GFP, which can express multiplex gRNAs (Hashimoto et al. 2018), to generate a *GuCSyGT*-targeting vector.

Due to the difficulty of generating transgenic licorice plants recalcitrant to *Agrobacterium tumefaciens*-mediated transformation, we used the hairy root transformation system. After introducing a *GuCSyGT*-targeting vector into *Agrobacterium rhizogenes* (ATCC15834), transgenic hairy roots were induced from licorice seedlings via infection with *A*. *rhizogenes* harboring *GuCSyGT*-targeting vector or the empty vector as a control.

Transgenic hairy roots were induced by culturing explants for 4–6 weeks and isolated. Mutations in the target regions were analyzed by a heteroduplex mobility assay (HMA) and sequence analysis. HMA analysis of 158 isolated hairy roots identified 47 potential genome-edited (GE) lines. Sequencing analysis of the target sites in these 47 lines identified two GE lines: GE-1 and GE-2 (Fig. 2b).

For line GE-1, 16 randomly selected clones were sequenced and a 403 bp deletion/23 bp insertion causing a frameshift mutation was detected in the region spanning from 4 bp upstream of Target 1 to 20 bp downstream of Target 4 (Fig. 2b), suggesting that the transgene-encoded Cas9 and gRNAs were able to efficiently induce double-strand breaks at the Target 1 and Target 4 sites in GE-1.

For line GE-2, seven randomly selected clones were sequenced and two different deletion patterns were detected (Fig. 2b). Three clones had a 364 bp deletion/21 bp insertion in the region spanning Targets 1–4. This deletion/insertion induced a 66-amino-acid deletion and an 8-amino-acid insertion. The remaining four clones had a 49 bp deletion in the region between the middles of Targets 1 and 2, resulting in a frameshift mutation. These results indicate that the transgene-encoded Cas9 and gRNAs were able to efficiently induce double-strand breaks at the Target 1 and Target 4 sites in allele 1 and the Target 1 and Target 2 sites in allele 2 in GE-2. We detected no intact *CSyGT* sequence in GE-1 or GE-2.

### Involvement of *GuCSyGT* in soyasaponin biosynthesis

While licorice hairy roots do not produce detectable levels of glycyrrhizin or the corresponding sapogenin glycyrrhetinic acid, licorice hairy roots produce detectable amounts of soyasaponin I as the predominant saponin. To examine the effects of the disruption of *CSyGT* on the biosynthesis of triterpenoid saponins, we analyzed the contents of soyasaponin I and its biosynthetic intermediates in the control and GE lines by liquid chromatography–mass spectrometry (LC–MS).

**Fig. 3:**
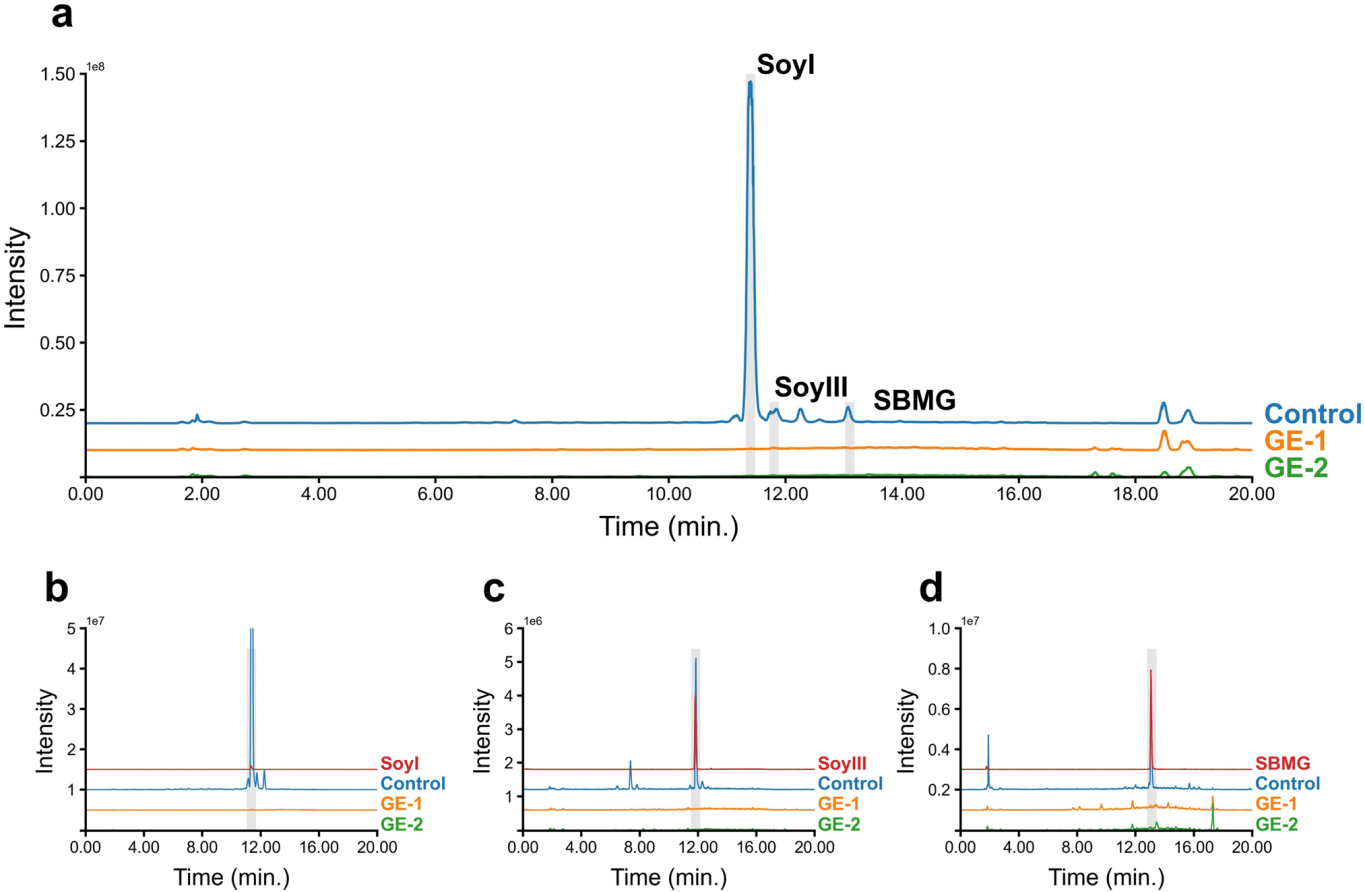
LC–MS analysis of soyasaponin I and its biosynthetic intermediates contained in the control and genome-edited hairy roots. **a** Total ion chromatograms (TICs) of saponins extracted from empty vector control (Control) and genome-edited (GE) lines. Selected ion monitoring of hairy roots and the authentic standards for *m/z* equivalent to **b** soyasaponin I (SoyI; 941.5), **c** soyasaponin III (SoyIII; 795.5), and **d** soyasapogenol B monoglucuronide (SBMG; 633.4)

As shown in Fig. 3 and Supplementary Table S3, soyasaponin I and its biosynthetic intermediates soyasaponin III and SBMG were completely absent in both GE-1 and GE-2, indicating that GuCSyGT plays an essential role in soyasaponin biosynthesis *in planta*. The absence of SBMG in the GE lines strongly supports our previous finding that GuCSyGT catalyzes the 3-*O*-glucuronosylation of soyasapogenol B (Chung et al. 2020).

**Fig. 4:**
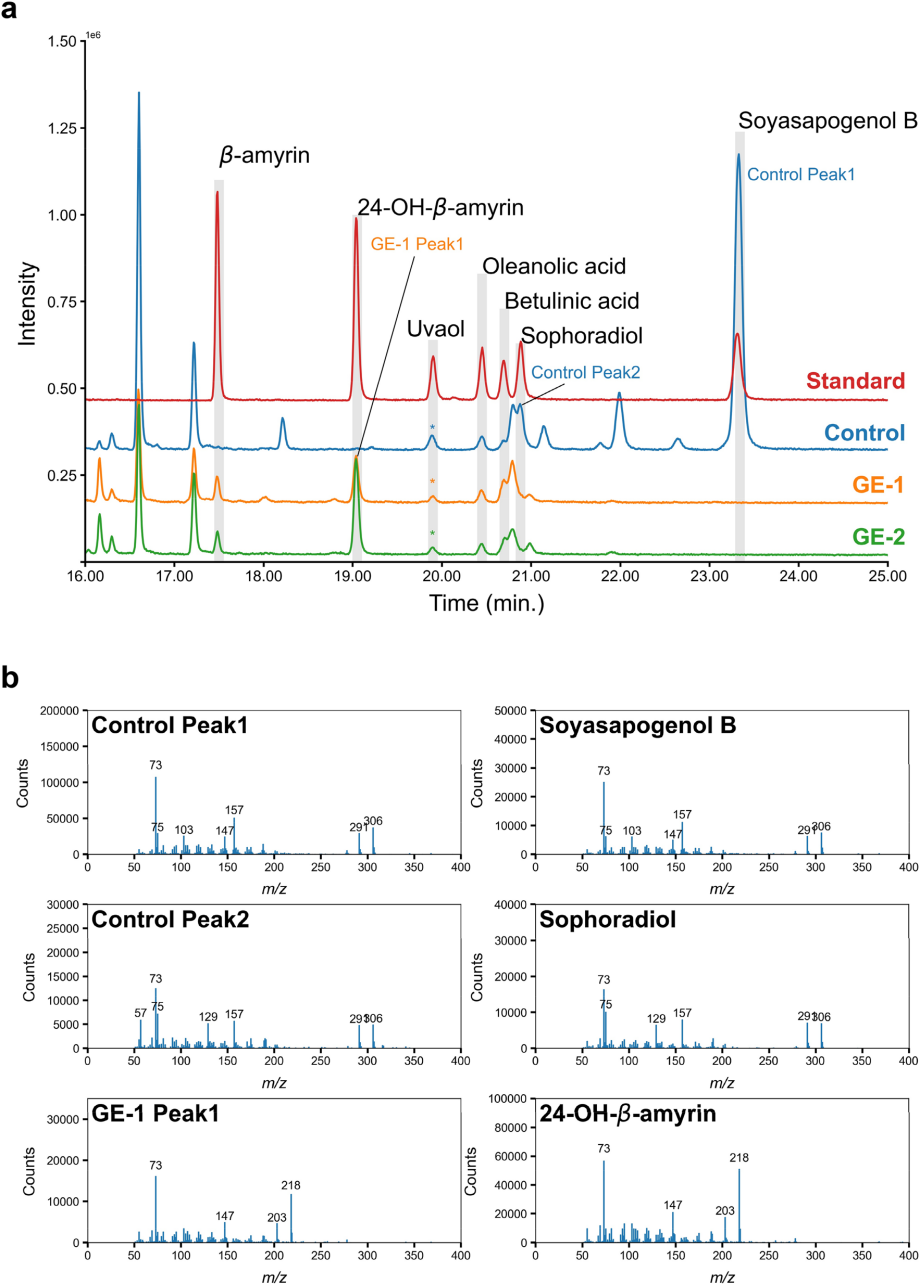
GC–MS analysis of acid-hydrolyzed extracts from control and genome-edited hairy roots. **a** Hairy root extracts from the empty vector control (Control) and genome-edited lines (GE-1 and GE-2) were compared to authentic standards (Standard). Uvaol (*) was used as an internal standard. **b** The MS spectra of Peak 1 and Peak 2 in the control line (Control Peak 1 and Control Peak 2) show excellent matching to those of authentic soyasapogenol B and sophoradiol, respectively. The MS spectrum of Peak 1 in GE-1 (GE-1 Peak 1) shows excellent matching to that of authentic 24-OH-β-amyrin. An analysis of other triterpenoids by MS is shown in Supplementary Figure S1

Upon inactivation of GuCSyGT, an increase in its substrate soyasapogenol B would be expected. To confirm that soyasapogenol B was synthesized and increased in the GE lines, we analyzed the sapogenins in acid-hydrolyzed extracts of the control and GE lines by gas chromatography-mass spectrometry (Fig. 4). Acid hydrolysis removed the sugar moieties from saponins; Fig. 4 shows the total amount of the non-glycosylated form and aglycone part of the saponins. Soyasapogenol B was detected in a control line (Control Peak 1 in Fig. 4a; the mass spectrum of the Control Peak 1 was an excellent match with that of authentic soyasapogenol B as shown in Fig. 4b). Unexpectedly, however, it was not detected in GE-1 or GE-2 (Fig. 4a; Supplementary Table S4). This result not only negates the possibility that soyasapogenol B was metabolized into saponins with a different sugar chain composition from that of soyasaponin I, but also indicates that soyasapogenol B biosynthesis was inhibited in GE lines. To obtain insight into the possible mechanisms underlying the absence of soyasapogenol B in GE lines, we also analyzed other triterpenoids as precursors of soyasapogenol B, including sophoradiol (22-hydroxy-β-amyrin), 24-hydroxy-β-amyrin, and β-amyrin, and other triterpenoids produced through competing pathways (Fig. 1a). Interestingly, GE-1 accumulated 24-hydroxy-β-amyrin (GE-1 Peak 1 in Fig. 4a), which was not detected in the control line. However, GE-1 lacked sophoradiol, which was detected in the control line (Control Peak 2 in Fig. 4a). Similar metabolite changes were observed in GE-2 (Fig. 4a). The mass spectra of GE-1 Peak 1 and Control Peak 2 showed excellent matches with those of authentic 24-hydroxy-β-amyrin and sophoradiol, respectively (Fig. 4b). The estimated content of each sapogenin is shown in Supplementary Table S4.

These results indicate that the CYP72A566-catalyzed conversion of β-amyrin and 24-hydroxy-β-amyrin to sophoradiol and soyasapogenol B, respectively, was hampered in GE lines by unknown mechanisms. We also observed a slight increase in betulinic acid in GE lines (Fig. 4a; Supplementary Fig. S4), suggesting that some of the oxidosqualene pool was redirected to the betulinic acid pathway.

## Discussion

**Fig. 5:**
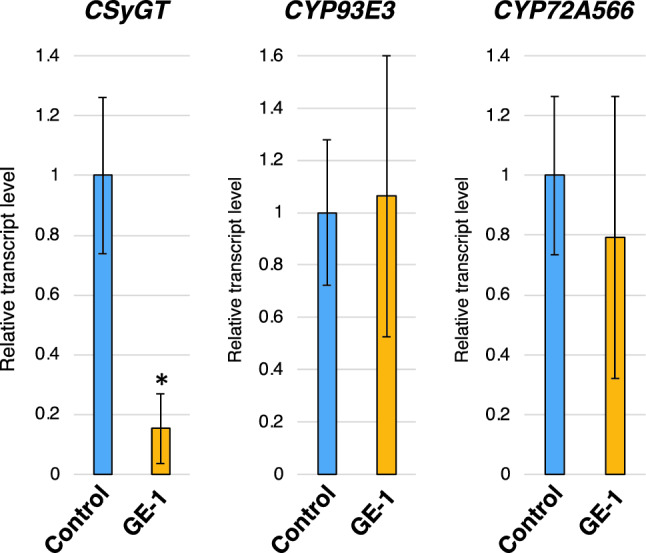
qRT-PCR analysis of *CYPs* and *CSyGT* genes in the control and GE-1.The values were normalized against the expression level of a reference gene (β-tubulin). Error bars indicate the standard deviation of three technical replicates. *P < 0.05

We generated *GuCSyGT*-disrupted licorice hairy roots by CRISPR–Cas9-mediated genome editing and revealed that soyasaponin I and its biosynthetic intermediates, soyasaponin III and SBMG, were completely absent from *GuCSyGT*-disrupted lines (Fig. 3). These results demonstrate that GuCSyGT plays an essential role in soyasaponin biosynthesis *in planta*. GuCSyGT catalyzed 3-*O*-glucuronosylation of soyasapogenol B when expressed in yeast (Chung et al. 2020), which led us to predict the accumulation of unglycosylated soyasapogenol B in *GuCSyGT*-disrupted hairy roots. Surprisingly, however, an analysis of acid-hydrolyzed extracts of the *GuCSyGT*-disrupted lines revealed an absence of soyasapogenol B (Fig. 4). Interestingly, this was accompanied by marked 24-hydroxy-β-amyrin accumulation and the absence of sophoradiol (Fig. 4). These results indicate that the CYP72A566-catalyzed conversion of β-amyrin and 24-hydroxy-β-amyrin to sophoradiol and soyasapogenol B, respectively, was hampered in *GuCSyGT*-disrupted lines via unknown mechanisms. We compared the *CYP72A566* transcript levels between the control and GE-1 by quantitative PCR (qPCR); however, the transcript levels did not differ significantly between the control and GE-1 as in the case of *CYP93E3* (Fig. 5), indicating that the absence of soyasapogenol B was not due to transcriptional repression of *CYP72A566*. We also analyzed the transcript levels of *GuCSyGT* in the control and GE-1. As shown in Fig. 5, a significant decrease in the *GuCSyGT* mRNA level was observed in GE-1 compared to the control line. This might be due to nonsense-mediated mRNA decay because the deletion that occurred in GE-1 generated many premature termination codons.

Marked 24-hydroxy-β-amyrin accumulation and the absence of sophoradiol in *GuCSyGT*-disrupted lines (Fig. 4) suggest that the preferred route of soyasaponin biosynthesis involves glucuronosylation at C3 of 24-hydroxy-β-amyrin followed by hydroxylation at C22 to produce SBMG (Fig. 1b); however, a detailed and comparative biochemical characterization of CYP72A566 using 24-hydroxy-β-amyrin and 24-hydroxy-β-amyrin 3-*O*-monoglucuronide as substrates is necessary to verify this speculation.

In summary, this study not only demonstrates the *in planta* role of GuCSyGT in soyasaponin biosynthesis, it also clearly shows the utility of genome editing for gene function analysis and metabolic engineering in hairy root cultures of non-model plants recalcitrant to *A. tumefaciens*-mediated transformation. Simultaneous disruption of the soyasaponin biosynthetic *CYPs CYP93E3* and *CYP72A566* might be an ideal approach for “metabolic switching” from the soyasaponin to glycyrrhizin pathway in the hairy roots of licorice.

### Supplementary Information

Below is the link to the electronic supplementary material.

Supplementary file1 (PDF 541 KB)

Supplementary file2 (DOCX 39 KB)

## Data Availability

The data supporting the findings of this study are available within the article and its supplementary material.
