# Supplementary material for: Disruption of a licorice cellulose synthase-derived glycosyltransferase gene demonstrates its in planta role in soyasaponin biosynthesis
Source: Plant Cell Rep. 2023 Dec 23;43(1):15. doi: 10.1007/s00299-023-03095-6 (PMC10746781; doi:10.1007/s00299-023-03095-6)
Supplement: Supplementary file 1 — Supplementary file1 (PDF 541 KB) [file 299_2023_3095_MOESM1_ESM.pdf]

Supplementary Table S1. Primers used in this study

Primer No.	Sequence (5′ to 3′)	comment
1	ATGGCAAGCTTCACCCTTCACACAGAAACC	cloning of *GuCSyGT*
2	TTATCCACTCTTGCTTTTCATGGTTATTATCC	cloning of *GuCSyGT*
3	TTGGGTCTCGTGCAGCCCGGCAGCTTCGCGTCGCTGTTTTAGAGCTAGAAATAGCA	*GuCSyGT* gRNA T1
4	TTGGGTCTCCTCAAGCGTGCACTGCACCAGCCGGGAATCGAA	*GuCSyGT* gRNA T2
5	TTGGGTCTCGTTGACCCCGAGAGTTTTAGAGCTAGAAATAGCA	*GuCSyGT* gRNA T2
6	TTGGGTCTCCACTCTTTACGGCTGCACCAGCCGGGAATCGAA	*GuCSyGT* gRNA T3
7	TTGGGTCTCGGAGTGACCGGGGGTTTTAGAGCTAGAAATAGCA	*GuCSyGT* gRNA T3
8	TTGGGTCTCCAAACCACCCTCGCGAACTCAGAAGCTGCACCAGCCGGGAATCGAA	*GuCSyGT* gRNA T4
9	CCTCGCAGTTGTACTGCTCCTCTTA	target site PCR Fw
10	GACCTGAACTCGTCGGTGCGAAGAA	target site PCR Rev
11	GCCGCCGATTACTTCTTCATTC	qPCR for *CYP93E3*
12	CGCGGATATTGACAAAGTGCTC	qPCR for *CYP93E3*
13	CAGGGGAGCCTAGTAACAATGAC	qPCR for *CYP72A566*
14	GCCCTGCCAAGTAAAATAGCTTC	qPCR for *CYP72A566*
15	GACCCTTGGTTTGCAGTGTT	qPCR for *GuCSyGT*
16	GATTGCGAACACGCTAGTGA	qPCR for *GuCSyGT*
17	TCTTCGCAAACTGGCAGTGA	qPCR for *β-tubulin*
18	CGAGATGTGAGTGGGGCAAA	qPCR for *β-tubulin*

Supplementary Table S2. gRNA target sequences

The underlines indicate PAM sequences.

No.	Sequence (5′ to 3′)
Target-1	CCCAGCGACGCGAAGCTGCCGGG
Target-2	GTGCACGCTTGACCCCGAGAAGG
Target-3	CCGCCCCGGTCACTCTTTACGGC
Target-4	CTTCTGAGTTCGCGAGGGTGTGG

Supplementary Table S3. Estimated concentration (μg/mg-dw) of soyasaponin I and its biosynthetic intermediates soyasaponin III and SBMG in hairy roots

Estimation was performed based on peak areas from the chromatogram shown in Figure 3, by comparing the peak area of the corresponding authentic standard with known concentrations. n.d., not detected.

Compound	**Control**	**GE-1**	**GE-2**
**Soyasaponin I**	12.3	n.d.	n.d.
**Soyasaponin III**	0.229	n.d.	n.d.
**SBMG**	1.23	n.d.	n.d.

Supplementary Table S4. Quantification of sapogenins in acid-hydrolyzed extracts of hairy roots

Quantification (ng/mg-dw) was performed based on peak areas from the mass chromatogram shown in Figure 4a. The concentrations of triterpenoid sapogenins in hairy roots were determined by comparison with authentic standard curves. n.d., not detected.

Compound	**Control**	**GE-1**	**GE-2**
**β-amyrin**	n.d.	75.9	59.8
**24-OH-β-amyrin**	n.d.	302.7	501.4
**Oleanolic acid**	398.6	372.4	355.1
**Betulinic acid**	407.2	574.4	488.0
**Sophoradiol**	650.7	n.d.	n.d.
**Soyasapogenol B**	2845.1	n.d.	n.d.

a

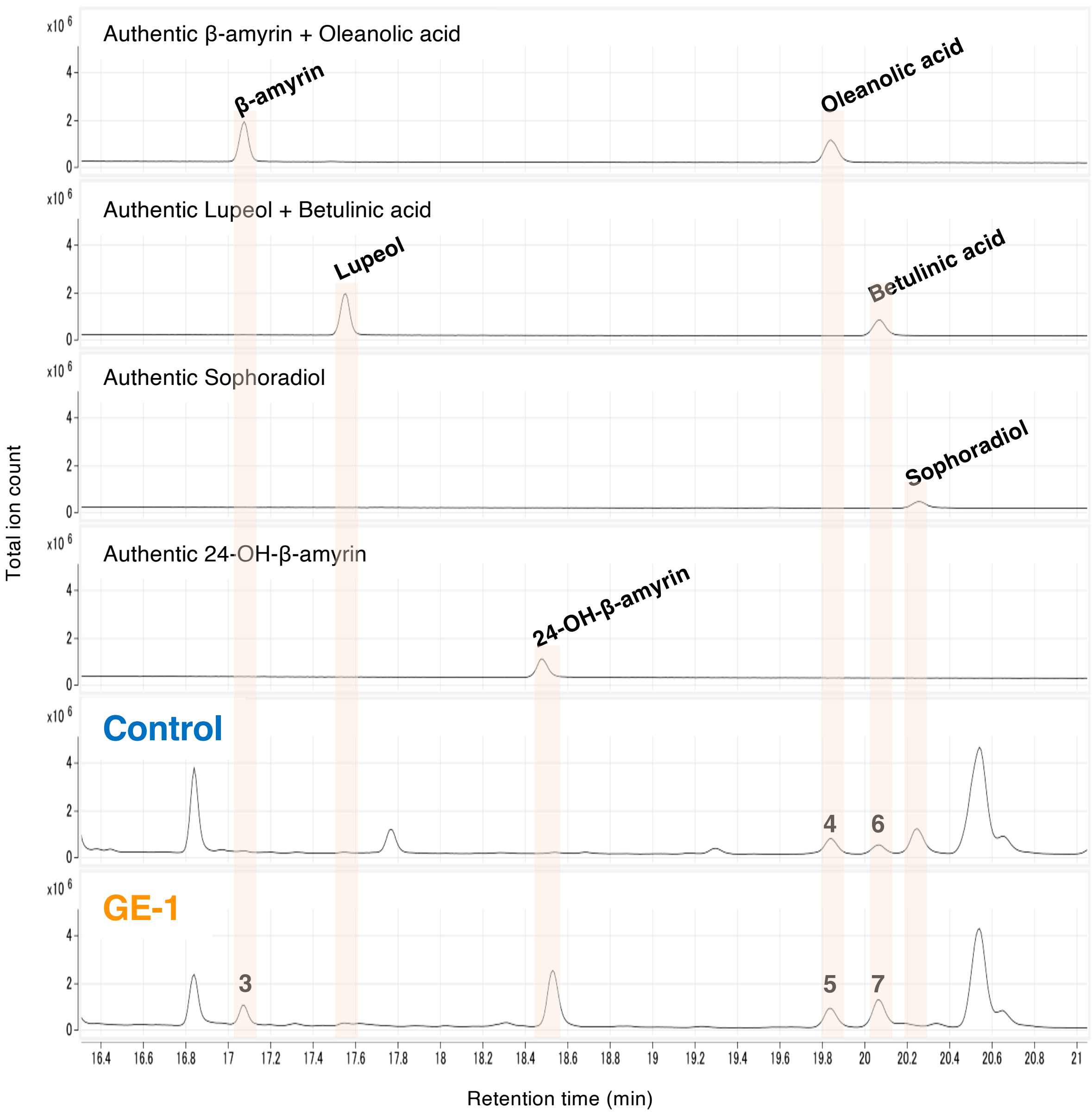

Sakanishi et al. Supplemerntary Figure S1

b

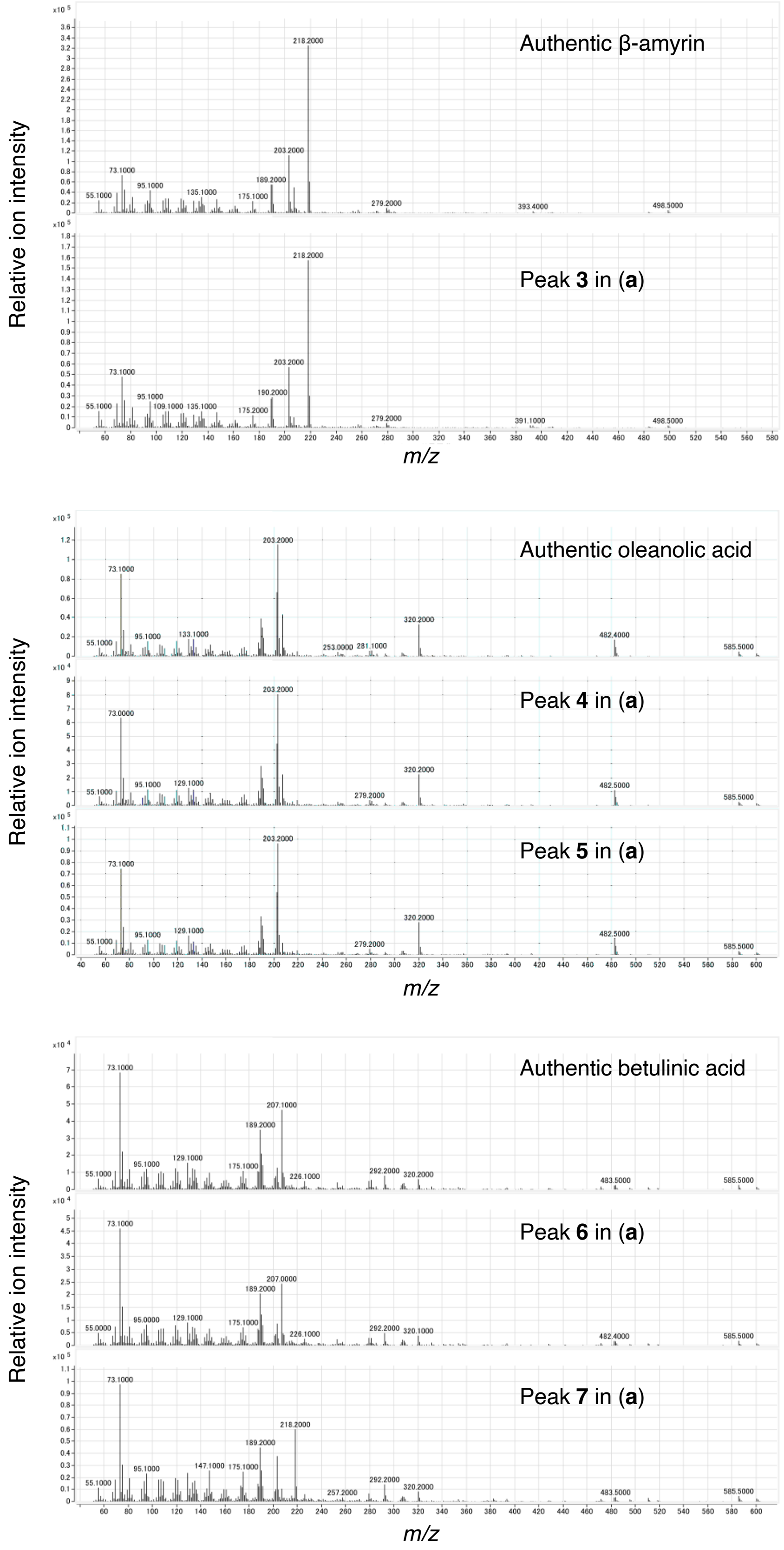

Sakanishi et al. Supplementary Figure S1 continued
