# Supplementary material for: Disruption of a licorice cellulose synthase-derived glycosyltransferase gene demonstrates its in planta role in soyasaponin biosynthesis
Source: Plant Cell Rep. 2023 Dec 23;43(1):15. doi: 10.1007/s00299-023-03095-6 (PMC10746781; doi:10.1007/s00299-023-03095-6)
Supplement: Supplementary file 2 — Supplementary file2 (DOCX 39 KB) [file 299_2023_3095_MOESM2_ESM.docx]

**Supplementary Table S1. Primers used in this study**

**Supplementary Table S2.** **gRNA target sequences**

**Supplementary Table S3. Estimated concentration (μg/mg-dw) of soyasaponin I and its biosynthetic intermediates soyasaponin Ⅲ and SBMG in hairy roots**

**Supplementary Table S4.** **Quantification of sapogenins in acid-hydrolyzed extracts of hairy roots**

**Supplementary Figure S1.** **GC profile of the acid-hydrolyzed extracts from the control and GE-1**

(a) TIC of the acid-hydrolyzed extracts from the control and GE-1 compared to authentic standards. (b) Mass spectra of peak 3 from the GC profile shown in (a) compared to that of authentic β-amyrin. Mass spectra of peaks 4 and 5 from the GC profile shown in (a) compared to that of authentic oleanolic acid. Mass spectra of peaks 6 and 7 from the GC profile shown in (a) compared to that of authentic betulinic acid.
